# SARC-F as a case-finding tool for sarcopenia according to the EWGSOP2. National validation and comparison with other diagnostic standards

**DOI:** 10.1007/s40520-020-01782-y

**Published:** 2021-01-28

**Authors:** Karolina Piotrowicz, Anna Głuszewska, Joanna Czesak, Małgorzata Fedyk-Łukasik, Ewa Klimek, Dolores Sánchez-Rodríguez, Anna Skalska, Barbara Gryglewska, Tomasz Grodzicki, Jerzy Gąsowski

**Affiliations:** 1 Department of Internal Medicine and Gerontology, Faculty of Medicine, Medical College, Jagiellonian University, Ul. Jakubowskiego 2, 30-688 Kraków, Poland; 2 Department of Internal Medicine and Geriatrics, University Hospital, Kraków, Poland; 3 Department of Clinical Rehabilitation, University School of Physical Education, Kraków, Poland; 4 Division of Public Health, Epidemiology and Health Economics, WHO Collaborating Centre for Public Health Aspects of Musculo-Skeletal Health and Ageing, University of Liège, Liège, Belgium; 5 Geriatrics Department, Rehabilitation Research Group, Hospital Del Mar Medical Research Institute (IMIM), Universitat Pompeu Fabra, Barcelona, Spain

**Keywords:** Sarcopenia, Screening, SARC-F, DXA, EWGSOP2

## Abstract

**Background:**

Sarcopenia is a potentially reversible condition, which requires proper screening and diagnosis.

**Aims:**

To validate a Polish version of sarcopenia screening questionnaire (SARC-F), and assess its clinical performance.

**Methods:**

Cross-sectional validation study in community-dwelling subjects ≥ 65 years of age. Diagnosis of sarcopenia was based on the 2018 2nd European Working Group on Sarcopenia in Older People (EWGSOP2) consensus. Hand grip and 4-m gait speed were measured, and the Polish version of SARC-F was administered.

**Results:**

The mean (SD) age of 73 participants (21.9% men) was 77.8 (7.3) years. Seventeen participants (23.3%) fulfilled the EWGSOP2 criteria of sarcopenia, and 9 (12.3%) criteria for severe sarcopenia. Fourteen (19.2%) participants fulfilled the SARC-F criteria for clinical suspicion of sarcopenia. The Cronbach’s alpha coefficient for internal was 0.84. With EWGSOP2 sarcopenia as a gold standard, the sensitivity of SARC-F was 35.3% (95% CI 14.2–61.7, *p* = 0.33), specificity was 85.7% (95% CI 73.8–93.6, *p* < 0.0001). The corresponding positive and negative predictive values were 42.9% (*p* = 0.79) and 81.4% (*p* < 0.0001), respectively. The probability of false-positive result was 14.3% (95% CI 6.4–26.2, *p* < 0.0001) and the probability of false-negative result was 64.7% (95% CI 38.3–85.8, *p* = 0.33). Overall the predictive power of SARC-F was low (c-statistic 0.64).

**Discussion:**

SARC-F is currently recommended for sarcopenia case finding in general population of older adults. However, its sensitivity is low, despite high specificity.

**Conclusions:**

At present SARC-F is better suited to rule out sarcopenia then to case-finding. Further refinement of screening for sarcopenia with the use of SARC-F seems needed.

**Supplementary Information:**

The online version contains supplementary material available at 10.1007/s40520-020-01782-y.

## Introduction

Sarcopenia is a frequent, age-related muscle wasting, that results in impaired skeletal muscle performance. Its prevalence has been estimated at 9–40% among older persons [1]. Sarcopenia has been linked to higher morbidity and mortality [2], increasing the need for support in the activities of daily living or institutionalization, and diminished quality of life [3, 4]. Sarcopenia is also responsible for high care-related burden, including burden to the family and the society [5, 6]. Sarcopenia is amenable to therapy, mainly rehabilitation and proper nutrition [7, 8]. However, protocols to assess sarcopenia are required, with sufficient performance in the case-finding and the confirmation stages of the diagnosis [9].

SARC-F is a simple, easy-to-use, 5-item sarcopenia screening questionnaire [10]. Since then, SARC-F has been translated and validated for use in different languages [11–16], various clinical settings [11, 13, 14, 17], and against various gold-standard diagnostic modalities then current [18, 19]. In 2018, SARC-F has been incorporated, as a sarcopenia case-finding tool, in the European Working Group on Sarcopenia in Older People 2 (EWGSOP2) diagnostic algorithm for sarcopenia [6].

However, the diagnostic performance of the test varies across studies [20]. As an example, the sensitivity of the test varies considerably across studies, with values ranging from 3.9 to 95.4% [21, 22].

Our aim, was to standardize the translation and validate the clinical performance of the Polish version, included in the on-line appendix, of the SARC-F as a case-finding tool against an array of objective diagnostic criteria for sarcopenia, including dual-energy X-ray absorptiometry (DXA)-based EWGSOP2.

## Methods

### Study

The study was approved by the Jagiellonian University Bioethics Committee (KBET no.: 1072.6120.71.2018). All participants gave written informed consent. The study was performed cross-sectionally between August 2018 and March 2019, in line with the call by the Special Interest Group (SIG) for Sarcopenia of the European Geriatric Medicine Society (EuGMS) for action to improve the screening and the diagnosis of sarcopenia [23].

### SARC-F questionnaire and other sarcopenia screening modalities

We performed a two-step cultural and clinical validation of SARC-F questionnaire in subjects ≥ 65 years of age. The validation protocol was based on the original validation procedure of the English SARC-F [23]. In brief, the questionnaire was translated from English to Polish by the two Polish geriatric researchers fluent in general and medical English (KP, AS). The final version of the Polish SARC-F was back-translated to English by a Polish-English bilingual native-speaker certified in interpreting between both languages. The back-translation was then sent to Prof. John Morley for the formal approval. The threshold for sarcopenia case-finding has been established at 4 points [10].

The original SARC-F questionnaire together with the Polish translation is included in Appendix 1 [10]. In addition to the SARC-F, we used the SARC-CalF which adds calf circumference to the SARC-F components [24]. We also used mid-arm circumference and calf circumference as separate screening instruments. For the calf and mid-arm circumference, we used population-specific cut-off values based on Youden’s Index. For the calculation SARC-CalF, on a scale from 0 to 20, we used the Youden Index-based population-specific cut off for calf circumference (< 32 cm) with a weight of 10. The cut-off for SARC-CalF was ≥ 11. We also used index by Ishii et al. [25].

### Study population

Information about the study was sent to the local seniors’ organizations (e.g. universities of the third age, senior clubs) and posted in the outpatient and inpatient geriatric clinics of the University Hospital in Kraków. Consecutive community-dwelling people aged 65 years and more were encouraged to participate in the project.

### Measures

Body composition was assessed by the Lunar iDXA dual-energy X-ray absorptiometry (DXA) equipment (GE Healthcare, Chicago, Il, USA). Appendicular lean body mass was calculated as a sum of lean mass of both upper and lower limbs, and expressed and further analyzed as Appendicular Skeletal Muscle Mass (ASM, kg), ASM adjusted for subject’s height (ASM/h^2^, kg/m^2^) and ASM adjusted for subject’s body mass index (BMI) (ASM/BMI) [6].

Muscle strength (kg) was assessed with a Saehan handheld dynamometer SH 5001 (Seahan Corporation, Masan, South Korea) according to the American Society of Hand Therapists (ASHT) recommendations [26]. Handgrip strength of both hands was assessed three times, and the highest value for either hand was recorded.

Gait speed (m/s) was measured three times, over a distance of 4 m with subjects walking at their usual speed, with walking aids if needed [27]. The first attempt was considered as an instructive example, and the highest value of the second or third trials was used.

Additionally, we performed standard Timed-Up and Go (TUG) Test and the Short Physical Performance Battery (SPPB) [28, 29]. The functional measurements were performed by a study physiotherapist (JC).

The subjects were interviewed by one of the two trained raters (KP, AG). To assess their cognitive performance we used Polish version of the Montreal Cognitive Assessment and 15-point Geriatric Depression Scale [30, 31], functional status the Activities of Daily Living and Instrumental Activities of Daily Living Scales and activity the Seven-Day Physical Activity Recall questionnaire [32, 33]. Physical frailty was assessed according to criteria by Fried et al. and Rockwood et al. [34, 35], the nutritional status with the Mini-Nutritional Assessment [36], and quality of life with the EuroQol-5D-5L questionnaire [37]. Sociodemographic characteristics and information about medications and comorbidities were collected. Based on the available data, the Charlson Index and total weekly energy expenditure were calculated for each subject [33, 38].

Anthropometric measures were performed in accordance with the Centres for Disease Control and Prevention (CDC) guidelines [39], and included: height (cm), weight (kg), waist and hip circumference (cm), calf circumference (CC, cmf) and midarm circumference (MAC, cm); Body Mass Index (kg/m^2^) and Waist-Hip Ratio (WHR) was calculated, respectively.

### Assessment of sarcopenia

Sarcopenia was diagnosed according to four definitions including: the European Working Group on Sarcopenia in Older People 2 (EWGSOP2, 2018) consensus (all sarcopenia, not limited to severe sarcopenia)[6], the Foundation for the National Institutes of Health (FNIH, 2014), based on weakness and low muscle mass [40], the International Working Group on Sarcopenia (IWGS, 2011) [41], and the Society of Sarcopenia, Cachexia and Wasting Disorders (SSCWD, 2011) criteria [42]. For the purpose of the presented analysis, we used the values of AMS adjusted to height (ASM/h^2^) of ≤ 6.0 kg/m2 for women and ≤ 7.0 kg/m^2^ for men, respectively, as proposed by the EWGSOP2 consensus paper [6]. The operational criteria used for sarcopenia diagnosis are summarized and presented in Appendix 2.

### Statistical analyses

The data management and the statistical analyses were performed with SAS 9.4 (SAS Institute Inc., Cary, NC, USA). The continuous variables were compared with standard normal Z-test or Wilcoxon’s test, normally and non-normally distributed variables, respectively. The proportions were compared with chi-square test. To assess the coherence of the Polish version of the SARC-F inventory, we calculated Cronbach’s alpha coefficient. Further, for each objective gold-standard diagnosis of sarcopenia as a binary outcome, with SARC-F as an ordinal explanatory variable on a scale from zero to ten, we fitted a logistic regression model based on which we obtained the Receiver Operating Characteristics (ROC) curve with the Area Under the Curve (AUC) as a measure of diagnostic performance. Using the approach described by Youden et al. [43] we obtained the population-specific cut off values for SARC-F. Further, based on the cut-off of 4 proposed by the EWGSOP2, and the calculated population-specific cut-off, we calculated the sensitivity, specificity, positive predictive value, negative predictive value and accuracy, with exact 95% Confidence Intervals. To put the performance of SARC-F in a wider context, we repeated the procedure for alternative case-finding tests described in the literature.

## Results

The mean (SD) age of 73 patients (78.1% women) was 77.8 (7.3) years. Of the entire group, 17 persons had sarcopenia based on the EWGSOP2 criteria, while 14 participants fulfilled the criteria for the SARC-F defined sarcopenia. Table 1, contains the characteristics of the study group. Overall, the included patients were multimorbid and taking multiple medications (median quantity of OTC preparations 2, median quantity of prescription preparations 7.5) irrespective of their sarcopenia status. Cognitive impairment was present in 61.1%, malnutrition or the risk of malnutrition were present in 32% and frailty based on Fried criteria in 29.2% of the participants. Patients affected by sarcopenia were older, and had lower educational status, and were affected with more diseases. They had lower BMI, including lower muscle and lower fat mass, lower mid-arm circumference and lower calf circumference. Patients with sarcopenia, in general, were characterized by lower self-reported physical activity, and worse values for physical performance and muscle strength indices (Table 1).

**Table 1:** Baseline Characteristics

	All (*n* = 73)	SARC-F		*p*	EWGSOP2 consenus statement		*p*
		Sarcopenia	No sarcopenia		Sarcopenia	No sarcopenia	
Age (years)	77.8 (7.3)	77.6 (8.3)	77.9 (7.1)	0.91	82.1 (9.2)	76.6 (6.1)	0.006
Women (%)	78.1	92.9	74.6	0.14	70.6	80.4	0.40
Education (years)	14.0 (3.2)	12.0 (3.2)	14.4 (3.1)	0.01	13.3 (3.3)	14.2 (3.2)	0.34
Financial status				0.09			0.25
Income insufficient (%)	38.9	64.3	32.8		29.4	41.8	
Living alone (%)	48.6	21.4	55.2	0.02	47.1	49.1	0.89
Walking assist device (%)	13.9	42.9	6.9	0.0005	29.4	9.1	0.04
Chronic diseases (number)	4.7 (2.6)	6.1 (2.8)	4.3 (2.5)	0.02	5.8 (2.4)	4.3 (2.6)	0.04
Charlson Index (points)	1 (0–4)	1.5 (0–4)	1 (0–5)	0.03	2 (0–9)	1 (0–4)	0.002
Medications (number)	7.5 (2–13)	8 (3–13)	7 (1–13)	0.65	8 (4–11)	7 (1–13)	0.97
OTC-drugs (number)	2 (0–4)	1.5 (0–3)	2 (0–4)	0.57	1 (0–3)	2 (0–4)	0.48
BMI (kg/m^2^)	27.0 (5.2)	25.9 (5.8)	27.3 (5.0)	0.36	23.3 (4.0)	28.2 (5.0)	0.0004
WHR	0.9 (0.1)	0.9 (0.1)	0.90 (0.1)	0.59	0.9(0.1)	0.9 (0.1)	0.59
ASMM (kg)	17.5 (3.9)	15.0 (3.1)	18.1 (3.8)	0.008	14.2 (2.4)	18.5 (3.7)	< 0.0001
Fat mass (kg)	26.1 (9.7)	22.5 (11.4)	27.0 (9.2)	0.12	19.7 (7.9)	28.1 (9.4)	0.001
ASMM/BMI [kg/(kg/m^2^)]	0.7 (0.1)	0.6 (0.1)	0.7 (0.1)	0.03	0.6 (0.1)	0.7 (0.1)	0.19
ASMM/h^2^ (kg/m^2^)	6.8 (1.2)	6.4 (1.3)	6.9 (1.1)	0.15	5.9 (0.8)	7.1 (1.1)	< 0.0001
MAC (cm)	29.9 (4.2)	28.7 (5.8)	30.1 (3.7)	0.37	26.7 (3.8)	30.8 (3.8)	0.0002
CC (cm)	36.7 (4.0)	34.0 (5.0)	37.4 (3.5)	0.003	33.1 (4.2)	37.9 (3.3)	< 0.0001
Hand grip strength (kg)	17.6 (9.4)	8.9 (6.9)	19.7 (8.8)	< 0.0001	11.7 (7.1)	19.4 (9.4)	0.003
Chair stand test (s)	12.8 (5.2)	17.8 (7.4)	12.0 (4.2)	0.04	15.6 (6.9)	12.0 (4.3)	0.02
Gait speed (m/s)	1.0 (0.3)	0.7 (0.3)	1.1 (0.3)	< 0.0001	0.8 (0.3)	1.1 (0.3)	0.002
SPPB (points)	9.5 (2.7)	6.4 (3.6)	10.2 (1.9)	0.002	7.7 (3.3)	10.0 (2.3)	0.002
TUG (s)	9.3 (4.6)	15.3 (7.1)	7.9 (2.1)	0.002	11.5 (5.6)	8.7 (4.2)	0.03
ADL (points)	5.9 (0.4)	5.6 (0.7)	6.0 (0.2)	0.13	5.7 (0.7)	6.0 (0.2)	0.01
IADL (points)	7.6 (1.4)	6.8 (2.1)	7.8 (1.1)	0.11	6.7 (0.7)	7.9 (0.5)	0.002
MoCA Test (points)	22.6 (4.8)	21.3 (4.5)	22.9 (4.9)	0.27	18.2 (5.9)	23.9 (3.6)	< 0.0001
Cognitive impairment				0.52			0.0003
MCI	37.5	35.7	37.3		29.4	40.0	
Dementia	23.6	35.7	22.0		58.8	12.7	
GDS Test (points)	3.7 (2.7)	5.1 (3.3)	3.4 (2.5)	0.03	3.7 (2.9)	3.7 (2.7)	0.99
Weight loss							
Last 3 months (kg)	0 (0–5)	1.3 (0–5)	0 (0–5)	0.04	0 (0–5)	0 (0–5)	0.38
Last 6 months (kg)	0 (0–10)	3 (0–10)	0 (0–10)	0.03	0 (0–5)	0 (0–10)	0.65
Nutritional status				0.27			0.09
Risk of malnutrition (%)	27.8	42.9	24.1		35.3	25.5	
Malnutrition (%)	4.2	7.1	3.5		11.8	1.8	
Frailty (Fried; %)	29.2	85.7	15.3	< 0.0001	52.9	21.8	0.02
Frailty (Rockwood; %)	15.3	57.1	5.1	< 0.0001	41.2	7.3	0.001
PA	4343.8 (2242.7–8951.6)	3038.1 (1508.8–17,484.2)	4352.0 (2488.5–8470.0)	0.11	3696.0 (1508.8–5390.0)	4874.3 (2346.0–9828.0)	0.001
EQ-5 (points)	0.8 (0.1)	0.7 (0.1)	0.9 (0.1)	< 0.0001	0.9 (0.1)	0.8 (0.1)	0.75
EQ-5 (%)	65.1 (15.4)	61.8 (16.0)	65.9 (15.3)	0.37	59.4 (14.7)	66.9 (15.3)	0.08
SARC-F							
Strength				< 0.0001			0.008
None	50.7	0.0	62.7		23.5	58.9	
Some	30.1	28.6	30.5		41.2	26.8	
A lot of/unable to do	19.2	71.4	6.8		35.3	14.3	
Assistance when walking				< 0.0001			0.06
None	80.8	7.1	98.3		64.7	85.7	
Some	15.1	71.5	1.7		29.4	10.7	
A lot of/unable to do	4.1	21.4	0.0		5.9	3.6	
Rise from a chair				< 0.0001			0.30
None	75.4	7.1	91.5		64.7	78.6	
Some	21.9	78.6	8.5		35.3	17.8	
A lot of/unable to do	2.7	14.3	0.0		0.0	3.6	
Climbing stairs				< 0.0001			0.37
None	64.4	21.4	74.6		58.8	66.1	
Some	24.6	21.4	25.4		17.7	26.8	
A lot of/unable to do	11.0	57.2	0.0		23.5	7.1	
Falls				0.0001			0.47
0	69.9	28.6	79.7		76.5	67.8	
1–3	27.4	57.1	20.3		23.5	28.6	
4 and more falls	2.7	14.3	0.0		0.0	3.6	

### Translation and cultural validation. Intra and interrater reproducibility

First, in an initial group of 10 persons of wide educational range (age 79.7 (9.3) years, 50% women) we assessed the ability by the participants to comprehend the questions correctly. We did that across different educational strata and across genders. Further, in another group of 20 participants we performed the assessment of the inter-rater and intra-rater agreement of each of the five items of the SARC-F test. Due to the fact that the answers were qualified into three levels, it was not possible to use the McNemar’s test. Instead, to check for the degree of agreement, we used the simple, unadjusted, kappa statistic. Overall, we found that the.

inter-rater agreement was high (the kappa statistics ranging between 0.85 and 1.0) and that the intra-rater reproducibility of the questions was good (the kappa statistics ranging between 0.65 and 1.0). (Appendix 3).

The Cronbach’s alpha test for internal consistency of questions (final study-group) was 0.82 for ability to lift and carry 10 lb, 0.83 for past year’s history of falls, 0.76 for chair to bed transfer, 0.76 for climbing 10 stairs and 0.74 for walking across a room. Overall, the Cronbach’s alpha was 0.82.

### Measures of clinical usefulness

To assess the clinical usefulness of SARC-F to detect the cases of sarcopenia, we used, as standards the following definitions of sarcopenia: EWGSOP2, IWGS, FNIH, and SSCWD. The sensitivity and specificity of SARC-F was 35.3% and 85.7% for EWGSOP2, 38.5% and 85.0% for IWGS, 30.0% and 82.5 for FNIH, 50.0% and 86.9% for SSCWD. The corresponding PPV and NPV were 42.9% and 81.4% for EWGSOP2, 35.7% and 86.4% for IWGS, 21.4% and 88.1% for FNIH, and 42.3 and 89.8% for SSCWD. The c-statistics for SARC-F were as follows: 0.64 for EWGSOP2, 0.60 for IWGS, 0.57 for FNIH, and 0.68 for SSCWD. The details are given in Table 2.

**Table 2:** Sarcopenia Screening Tools validated against Sarcopenia Consensus Definitions

		Sensitivity (%)		Specificity (%)		PPV (%)		NPV (%)		Accuracy		c-statistics
EWGSOP2	17 (23.3)											
SARC-F		35.3	14.2–61.7	85.7	73.8–93.6	42.9	17.7–71.1	81.4	69.1–90.3	74.0	62.4–83.6	0.64
SARC-F ≥ 5		35.3	14.2–61.7	89.3	78.1–96.0	50.0	21.1–78.9	82.0	70.0–90.6	76.7	65.4–85.8	
SARC-CalF ≥ 11		41.2	18.4–67.1	98.2	90.5–100.0	87.5	47.4–99.7	84.6	73.5–92.4	84.9	74.6–92.2	0.72
Ishii tool ≥ 136		88.2	63.6–98.5	75.0	61.6–85.6	51.7	32.5–70.6	95.5	84.5–99.4	78.1	66.9–86.9	0.88
CC < 32		41.2	18.4–67.1	98.2	95.0–100.0	87.5	47.4–99.7	84.6	73.5–92.4	84.9	74.6–92.2	0.81
MAC < 29		70.6	44.0–89.7	69.6	55.9–81.2	41.4	23.5–61.1	88.6	75.4–96.2	69.9	58.0–80.0	0.78
IWGS	13 (17.8)											
SARC-F		38.5	13.9–68.4	85.0	73.4–92.9	35.7	12.8–64.9	86.4	75.0–94.0	76.7	65.4–85.8	0.60
SARC-F ≥ 5		38.5	13.9–68.4	88.3	77.4–95.2	41.7	15.2–72.3	86.9	75.8–94.2	79.5	68.4–88.0	
SARC-CalF ≥ 11		61.5	31.6–86.1	88.3	77.4–95.2	53.3	26.6–78.7	91.4	81.0–97.1	83.6	73.1–91.2	0.83
Ishii tool ≥ 136		84.6	54.6–98.1	70.0	56.8–81.2	37.9	20.7–57.7	95.5	84.5–99.4	72.6	60.9–82.4	0.78
CC ≤ 35		76.9	46.2–95.0	86.7	75.4–94.1	55.6	30.8–78.5	94.6	84.9–98.9	84.9	74.6–92.2	0.91
MAC < 28		84.6	54.6–98.1	76.7	64.0–86.6	44.0	24.4–65.1	95.8	85.8–99.5	78.1	66.9–86.9	0.85
FNIH	10 (13.7)											
SARC-F		30.0	6.7–65.3	82.5	70.9–91.0	21.4	4.7–50.8	88.1	77.0–95.1	75.3	63.9–84.7	0.57
SARC-F ≥ 2		60.0	26.2–87.8	61.9	48.8–73.9	20.0	7.7–38.6	90.7	77.9–97.4	61.6	49.5–72.8	
SARC-CalF ≥ 11		60.0	26.2–87.8	65.1	52.0–76.7	21.4	6.2–36.6	91.1	78.8–97.5	64.4	52.3–75.3	0.69
Ishii tool ≥ 136		90.0	55.5–99.8	68.3	55.3–79.4	31.0	15.3–50.8	97.7	88.0–99.9	71.2	59.5–81.2	0.81
CC ≤ 37		80.0	44.4–97.5	54.0	40.9–66.6	21.6	9.8–38.2	94.4	81.3–99.3	57.5	45.4–69.0	0.73
MAC < 24		20.0	2.5–55.6	92.1	82.4–97.4	28.6	3.7–71.0	87.9	77.5–94.6	82.2	71.5–90.2	0.55
SSCWD	12 (16.4)											
SARC-F		50.0	21.1–78.9	86.9	75.8–94.1	42.3	17.7–71.1	89.8	79.2–86.2	80.8	69.9–89.1	0.68
SARC-F ≥ 5		50.0	21.1–78.9	90.2	79.8–96.3	50.0	21.1–78.9	92.0	79.8–96.3	83.6	73.1–91.2	
SARC-CalF ≥ 11		66.7	34.9–90.1	100.0	94.1–100.0	100.0	63.1–100.0	93.9	85.0–98.3	94.5	86.6–98.5	0.81
Ishii tool ≥ 154		66.7	34.9–90.1	86.9	75.8–94.2	50.0	24.7–75.4	93.0	83.0–98.1	83.6	73.1–91.2	0.79
CC ≤ 32		66.7	34.9–90.1	98.4	91.2–100.0	88.9	51.8–99.7	93.8	84.8–98.3	93.2	84.7–97.7	0.91
MAC < 28		91.7	61.5–99.8	77.1	64.5–86.9	44.0	24.4–65.1	97.9	88.9–100.0	79.5	68.4–88.0	0.90

In addition to the SARC-F, we used the SARC-CalF including calf circumference on top of the SARC-F components as described by Barbosa-Silva et al. [24] and a point scoring system designed by Ishii et al. [25] using sex, age, grip-strength, and calf-circumference

We also used midarm circumference and calf circumference as separate screening instruments. For the Ishii et al.’s index, the calf and midarm circumference, we used population-specific cut-off values based on Youden’s Index. For the calculation SARC-CalF, on a scale from 0 to 20, we used the Youden Index-based population-specific cut off for calf circumference (< 32 cm) with a weight of 10. The cut-off for SARC-CalF was ≥ 11

### Sample-specific cut-off values for SARC-F

Based on the ROC results for each of the sarcopenia definition used, we calculated sample-specific cut-off values of SARC-F. The Youden method-based cut-off with each sarcopenia standard as comparator was ≥ 5, except for FNIH where it was ≥ 2. The sensitivity of thus obtained cut-off against the FNIH as comparator was 60.0%, specificity was 61.9%, PPV was 20.0%, NPV was 90.7%. The sensitivity of this cut-off against the EWGSOP2 for was 35.3%, specificity was 89.3%, PPV was 50.0%, NPV was 82.0%. The corresponding values for the remaining comparator definitions of sarcopenia were not materially altered in comparison to standard SARC-F cut off. The details are given in Table 2.

### Other screening tools for sarcopenia

To put the clinical validity of SARC-F in a broader context, we analyzed the clinical validity of other screening modalities for sarcopenia.

With an exception of mid-arm circumference alone against FNIH as standard, all additionally tested screening criteria demonstrated numerically better c-statistic and sensitivity compared to SARC-F. The results for specificity, PPV, NPV, and accuracy varied and are presented in Table 2.

## Discussion

We performed a two-step, cultural and clinical validation of the Polish translation of the SARC-F questionnaire. We did that in the community-dwelling older persons, against the four commonly used definitions of sarcopenia. We used whole-body DXA scans to assess muscle mass.

SARC-F questionnaire was reproduceable both intra-rater (all kappa ≥ 0.82) and inter-rater (all kappa ≥ 0.62). The sensitivity and specificity of SARC-F against the EWGSOP2 was 35.3%, and 85.7%, respectively. We used the Youden method to obtain population-specific cut-off for SARC-F. This did not importantly improve the estimates of clinical validity.

SARC-F has been designed as a rapid screening tool for sarcopenia [10]. The SARC-F questionnaire has been widely translated and validated in specific populations [11–16, 22, 44, 45]. For some languages, the validation was performed in several populations, sometimes yielding conflicting results [12, 13, 46, 47].

In Poland, a recent study of 67 community-dwelling persons ≥ 65 years of age, the sensitivity of SARC-F was 92.9%, and the specificity 98.1% [47]. This contrasts results of similar another Polish study, where the sensitivity of SARC-F was 41.2%, specificity was 88.0% [46]. Both studies, used the bio-impedance weighting scale-measurement, however, the former one was performed in a slightly younger population (69.5 ± 4.0 years vs. 74.5 ± 6.9 years), which to some extend may have influenced the results. Based on DXA assessment, we show the sensitivity to be even smaller, with comparable specificity. A German validation study performed in 117 older subjects showed that the internal consistency of the German version of SARC-F was acceptable (the Cronbach alpha = 0.67), with intra-rater repeatability of 0.90 and inter-rater repeatability of 0.93. They estimated the sensitivity of SARC-F as 63%, specificity as 47% and the c-statistic of 0.58 [48]. Our estimates of the inter-rater and intra-rater repeatability for test components were > 0.85, and > 0.65, respectively. The overall Cronbach alpha was 0.82. The sensitivity was 35% and specificity 86%. The c-statistic was 0.64.

A number of studies, in persons of varied background including ethnicity, age, pathology were performed, yielding wide range of estimates of sensitivity, specificity, accuracy, and the ROC for SARC-F [20].

The largest study thus far was performed in 4000 older individuals from three populations of varied cultural background, demonstrated low sensitivity, but high specificity of SARC-F (< 10%, > 94%, gender-specific sensitivity and specificity, respectively). The authors of that report concluded that despite the low sensitivity and thanks to high specificity SARC-F may be used as a screening tool for sarcopenia at the community level [49].

SARC-F was employed in a range of pathologic settings. In the heart failure patients, the sensitivity and specificity were 52.5% and 96.2%, respectively [44]. In hip fracture patients, sensitivity was 95% and specificity 57%, [20] and in older orthopedic patients sensitivity was 47.4% and specificity 68.4% [50].

Disparate results of the psychometric characteristics of the SARC-F screening questionnaire demonstrated across sarcopenia studies, might be due to the varying diagnostic modalities used. As shown by Kim et al. in their study of 2099 community-dwelling older adults from the nationwide Korean Frailty and Aging Cohort Study (KFACS), sarcopenia prevalence varied from 7.9% if employing the chair stand test as a measure of muscle strength and ASM/height^2^ as a quantity unit for muscle mass, to 18.4% when handgrip strength and/or chair stand test and ASM/height^2^ results were examined [51].

In our study, we checked the diagnostic validity of the SARC-F against four definitions of sarcopenia (EWGSOP2, FNIH, IWGS, SSCWD expert guidelines). With grip strength as a measure of muscle strength, gait speed as a proxy of physical performance, and appendicular lean body mass checked with whole-body DXA scans, we showed an acceptable accuracy of the SARC-F for finding sarcopenia according to all the definitions tested. Additionally, we calculated the Youden’s J statistic and constructed the population-specific cut-off values for SARC-F against the EWGSOP2, FNIH, IWGS and SSCWD sarcopenia working group diagnostic criteria. We showed a sensitivity of SARC-F ranged from 30 to 50% and specificity from 83 to 87%, the results by and large in line with previously published data [18]. When setting the Polish population-specific cut off values for SARC-F questionnaire, we obtained better diagnostic properties when adjusting the threshold for suspected sarcopenia to five points for the EWGSOP2, IWGS and SSCWD, and two points for FNIH sarcopenia diagnostic consensus accordingly.

### The clinical performance of other screening tests

We found the SARC-F tool to be fairly specific but with low sensitivity. To put that in a broader context, we checked the clinical validity of other sarcopenia screening tools with our study population-specific cut-offs. The tools included: an index designed by Ishii et al. [25], calf circumference alone, midarm circumference alone, SARC-CalF – an index based on SARC-F that incorporates calf circumference [24], and the SARC-F with the study population-specific cut-off values. We found that for most of those tools the sensitivity was numerically better than for SARC-F. We also noted that the very simple measures such as calf circumference, or the midarm circumference were characterized by best clinical performance.

### Limitations and strengths

Our study needs to be considered in the context of its limitations. Our sample was moderate in size. However, its size was in line with what has been published thus far. We tested the SARC-F against an array of standards, where for the quantification of the muscle mass we used DXA. This may be an advantage over the studies that had used a bio-impedance based assessment of muscles.

## Conclusions and implications

We present a validated Polish translation of the SARC-F questionnaire. Although some other simple measures such as the mid-arm circumference or the calf circumference are at least of comparable value, SARC-F is more versatile, as it can be self-administered, assessed during a telephone interview, or used in subjects of varying body-build, or body-build affected by pathologies such as heart failure, liver failure, hypoalbuminemia etc. Our results indicate that its performance is better in ruling sarcopenia out than finding the cases.

## Supplementary Information

Below is the link to the electronic supplementary material.

Supplementary file1 (DOCX 18 KB)

## Data Availability

Available upon reasonable request. Analysis was based on SAS Base procedures.
