# Supplementary material for: SARC-F as a case-finding tool for sarcopenia according to the EWGSOP2. National validation and comparison with other diagnostic standards
Source: Aging Clin Exp Res. 2021 Jan 28;33(7):1821–9. doi: 10.1007/s40520-020-01782-y (PMC8249283; doi:10.1007/s40520-020-01782-y)
Supplement: Supplementary file 1 — Supplementary file1 (DOCX 18 KB) [file 40520_2020_1782_MOESM1_ESM.docx]

On-line appendices 1-3

SARC-F is a screening tool for sarcopenia. We found it’s Polish translation intra, and inter-rater reproducible, with low sensitivity and good specificity vs. the EWGSOP2 definition of sarcopenia, with DXA quantification of muscle.

Disclosures: None

| Component | Question | Scoring |
| --- | --- | --- |
| Strength | How much difficulty do you have in lifting and carrying 10 pounds? | None = 0  Some = 1  A lot or unable = 2 |
| Assistance in walking | How much difficulty do you have walking across a room? | None = 0  Some = 1  A lot, use aids, or unable = 2 |
| Rise from a chair | How much difficulty do you have transferring from a chair or bed? | None = 0  Some = 1  A lot or unable without help = 2 |
| Climb stairs | How much difficulty do you have climbing a flight of 10 stairs? | None = 0  Some = 1  A lot or unable = 2 |
| Falls | How many times have you fallen in the past year? | None = 0  1–3 falls = 1  4 or more falls = 2 |
| Komponenta | Pytanie | Punktacja |
| Siła | Jak dużą trudność sprawia Panu/Pani podnoszenie  i przenoszenie ciężaru około 5 kg? | Żadną trudność = 0  Pewną trudność = 1  Dużą trudność lub jest to niemożliwe = 2 |
| Pomoc przy chodzeniu | Jak dużą trudność sprawia Panu/Pani przejście przez pokój? | = 0  Pewną trudność = 1  Dużą trudność, wymaga użycia sprzętu pomocnicznego lub jest to niemozliwe = 2 |
| Wstawanie  z krzesła | Jak dużą trudność sprawia Panu/Pani przemieszczanie się  z krzesła lub z łóżka? | Żadną = 0  Pewną trudność = 1  Dużą trudność lub jest to niemożliwe bez pomocy = 2 |
| Wchodzenie  po schodach | Jaką ma Pan/Pani trudność  z wejściem po 10 schodach? | Żadną = 0  Pewną trudność = 1  Dużą trudność lub jest to niemożliwe = 2 |
| Upadki | Ile razy zdarzyło się Panu/Pani upaść w ciągu ubiegłego roku? | 0 upadków/wcale = 0  1-3 upadki = 1  4 upadki lub więcej = 2 |

Appendix1. The original (English)^10^ and the Polish SARC-F questionnaire.

| Operational definitions for sarcopenia | | | | |
| --- | --- | --- | --- | --- |
|  | | Muscle Strength | Muscle Mass | Physical Performance |
| European Working Group on Sarcopenia in Older People 2 (EWGSOP 2)^6^ | Sarcopenia | Handgrip strength:  Men < 27 kg  Women < 16 kg | Appendicular skeletal muscle mass:  Men < 20 kg  Women < 15 kg | ___ |
|  | Severe sarcopenia | Handgrip strength:  Men < 27 kg  Women < 16 kg | Appendicular skeletal muscle mass:  Men < 20 kg  Women < 15 kg | Gait speed:  ≤ 0.8 m/s |
| Foundation for the National Institutes of Health (FNIH) Sarcopenia Project ^40^ | Weakness and low muscle mass | Handgrip strength:  Men < 26 kg  Women < 16 kg | Appendicular skeletal muscle mass  adjusted to BMI:  Men < 0.789  Women < 0.512 | ___ |
|  | Slowness with weakness and low muscle mass | Handgrip strength:  Men < 26 kg  Women < 16 kg | Appendicular skeletal muscle mass  adjusted to BMI:  Men < 0.789  Women < 0.512 | Gait speed:  ≤ 0.8 m/s |
| International Working Group on Sarcopenia (IWGS) ^41^ |  | ___ | Appendicular skeletal muscle mass  adjusted to height^2^:  Men ≤ 7.23 kg/m^2^  Women ≤ 5.67 kg/m^2^ | Gait speed:  < 1.0 m/s |
| Society of Sarcopenia,  Cachexia and  Wasting Disorders (SSCWD) ^42,23^ |  | ___ | Appendicular skeletal muscle mass  adjusted to height^2^:  > of 2 SD below the mean of healthy  persons aged 20–30 years of the same  ethnic group*^6^  Men ≤ 7.0 kg/m^2^  Women ≤ 6.0 kg/m^2^ | Gait speed:  ≤ 1.0 m/s |

Appendix 2. Summary of the operational criteria used for sarcopenia diagnosis.

* values of AMS adjusted to height (ASM/h^2^) corresponding to the values of > 2 SD below the mean of healthy persons aged 20–30 years of the same ethnic group were retrieved from the EWGSOP2 consensus paper.^6^

| Q | Kappa (r1 vs r2) | LCL | UCL |
| --- | --- | --- | --- |
| S1 | 0.85 | 0.65 | 1.0 |
| S2 | 1.0 | 1.0 | 1.0 |
| S3 | 1.0 | 1.0 | 1.0 |
| S4 | 0.91 | 0.75 | 1.0 |
| S5 | 1.0 | 1.0 | 1.0 |
|  | | | |
| Q | Kappa (r1 vs r1  re-assessment) | LCL | UCL |
| S1 | 0.85 | 0.65 | 1.0 |
| S2 | 0.77 | 0.35 | 1.0 |
| S3 | 0.62 | 0.29 | 0.95 |
| S4 | 0.81 | 0.56 | 1.0 |
| S5 | 1.0 | 1.0 | 1.0 |

Appendix3.Simple (unadjusted) kappa coefficients* ± 95% CI for interrater and intra-rater agreement,

respectively, across the 5 questions included in SARC-F.

*Note: The answers to questions have three levels, hence the McNemar’s test is not applicable.
